# The relationship between big five personality and quality of life of people with disabilities: The mediating effect of social support

**DOI:** 10.3389/fpsyg.2022.1061455

**Published:** 2023-01-05

**Authors:** Lin Cai, Jiaxin He, Yibo Wu, Xuji Jia

**Affiliations:** ^1^School of Marxism, Sichuan Institute of Industrial Technology, Deyang, China; ^2^School of Public Health, Peking University, Beijing, China; ^3^Faculty of Psychology, Tianjin Normal University, Tianjin, China; ^4^Academy of Psychology and Behavior, Tianjin Normal University, Tianjin, China

**Keywords:** big five personality, quality of life, social support, people with disabilities, ecological systems theory

## Abstract

**Objective:**

The quality of life of people with disabilities is of great significance to social stability and development. Increasing the quality of life among the disabled has become a worldwide topic. This study aims to examine the relationship between the big five personality and quality of life and the mediating effects of social support indicators in people with disabilities.

**Methods:**

This was a cross-sectional study with 358 people with disabilities (193 women and 165 men). A questionnaire was utilized to measure big five personality, social support, and quality of life variables. Pearson’s correlation analysis and structural equation modeling were used to examine the relation among big five personality, social support, and quality of life.

**Results:**

QOL was positively correlated with social support (*r* = 0.402*, p* < 0.001), extraversion (*r* = 0.324*, p* < 0.001), agreeableness (*r* = 0.474*, p* < 0.001), conscientiousness (*r* = 0.482*, p* < 0.001), and openness (*r* = 0.498*, p* < 0.001). QOL was negatively correlated with neuroticism (*r* = −0.186*, p* < 0.001). The mediating effect of social support on the relationship between neuroticism and the quality of life of people with disabilities was not significant. Social support significantly mediated the relationship between extraversion, agreeableness, conscientiousness, openness, and quality of life. Overall, positive personality traits (extraversion, agreeableness, conscientiousness, and openness) in the Big Five Personality of people with disabilities could increase their quality of life by Perceiving social support. But social support could not significantly mediate the relationship between neuroticism and the quality of life of people with disabilities.

**Conclusion:**

These new findings suggest that combining individual factors (personality) and environmental factors (social support) can better improve the quality of life of people with disabilities.

## Introduction

The ultimate goal of social development is to improve the quality of human life. With the continuous progress of society, people are pursuing a higher quality of life (QOL) while ensuring the right to survival and development. How to improve the quality of human life is a highly important topic in the fields of social medicine and psychology. The research on QOL in social medicine has experienced three periods: First, in the early stage of the study, the study of QOL originated in the United States in the 1930s and was first used as a sociological indicator. The second is the mature period. The 1950s to 1960s were the rising period of life quality research. Since the publication of the anthology of social indicators edited by Bauer (1996), there have been two major schools in the field of sociological indicators: one is the objective sociological indicator school, which mainly reflects the level of social development by using some social and environmental objective condition indicators. The second is the school of subjective QOL, which emphasizes the subjective feelings of individuals toward society and the environment. The third is the differentiation period, which is the peak period of life quality research in the field of sociology, and gradually formed a research boom.

World Health Organization data report that there are more than 1 billion people worldwide with some form of disability, and the number of people with disabilities (PWD) is still increasing (The Lancet, 2011). PWD are more likely to be limited in their daily lives, so all aspects increase the risk of death for the disabled without knowing it. Research in China has indicated that the life expectancy of PWD is significantly lower than those of the general population (Zheng and Chen, 2011). The physical disability of the disabled is a significant cause that prevents them from improving their QOL (Chaudhary et al., 2019). Other country studies found that the mortality rate of PWD was one time higher than those without disabilities (Bahk et al., 2022), and people with multiple disabilities had the highest mortality rate (Forman-Hoffman et al., 2015). As a special vulnerable and marginalized group, the QOL of disabled people deserves our attention. The research on the influencing factors of their QOL will help improve their QOL, improve their happiness, and also improve the level of social civilization.

## Theory and hypotheses

### Big five personality and quality of life

McKeon believes that the concept of QOL can be traced back to Aristotle’s relevant argument that happiness represents a good life (Zhan, 1992). But it was not until after the second world war that the name “quality of life” was widely used. In the initial stage, the QOL only refers to good “material life,” after which scholars add the meaning of “psychological” or “social level” (Farquhar, 1995). The healthcare field began to pay attention to the importance of QOL, which can be traced back to the World Health Organization proposing in 1947 that the definition of health is not only the absence of disease, but also the physical, psychological, and social well-being. However, it was not until the 1960s that the concept of QOL was widely used in health research (Renzaho et al., 2016). In addition, early studies often used the concept of QOL with terms such as happiness, morale, or life satisfaction (Kuyken et al., 1995). However, this practice was later questioned by scholars, who pointed out that these terms are not equivalent to the concept of QOL (Lau and Mckenna, 2001).

In order to clarify the essence of the concept of QOL, scholars have tried to define QOL from subjective, objective, or multi-level perspectives. For example, Sarvimäki and Stenbock-Hult (2000) define the QOL as the subjective feeling of an individual’s health, value, or meaning. Lawton (1991) thinks that QOL is a multi-faceted concept, including objective environment, behavioral ability, QOL understood by individuals, and mental health. Zhan (1992) defined the QOL as: the degree of personal satisfaction with life experience, which includes four factors such as life satisfaction, self-concept, health and function, and socio-economic status. According to the 1995 WHO definition, QOL refers to individual culture and value systems, and experience of survival status related to people’s goals, expectations, standards, and concerns. It is a subjective assessment (Harper and Power, 1998). QOL contains multidimensional and subjective components, so individual, family, social, and other environmental factors will affect the QOL (Dijkers, 1999).

The bio-psycho-social model posits that in addition to biological factors such as bacteria and viruses, some psychological factors can also affect the health status of the human body to a certain extent (Liang et al., 2006; Du, 2009). Personality is one of the important psychological factors affecting the QOL (Diener et al., 1999; Jaiswal et al., 2018). The “Big Five” personality model believes that human personality traits consist of five traits: neuroticism, extraversion, agreeableness, conscientiousness, and openness. The diverse strength of each personality trait can cause different effects on various aspects of the human body (Sutin et al., 2013). Previous studies show that personality traits are closely related to diseases (Cao and Liu, 2017), and personality traits can predict human health outcomes (Yao et al., 2018). Many studies have confirmed that agreeableness (Rassart et al., 2013; Fuente-Arias et al., 2020), conscientiousness (Warrian et al., 2009), and openness (Goodwin and Engstrom, 2002) positively predicted the QOL. Neuroticism can negatively predict the QOL (Harandi et al., 2020; Dedova et al., 2022). Few studies showed that extraversion negatively predicted the QOL (Warrian et al., 2009; Harandi et al., 2020). The vast majority of studies showed that extraversion positively predicted the QOL (Goodwin and Engstrom, 2002; Masthoff et al., 2007; Poppe et al., 2013; Ibrahim et al., 2015). Based on the existing studies, the present study proposed the following hypotheses:

*H1a*: Neuroticism negatively predicts QOL.

*H1b*: Extraversion positively predicts QOL.

*H1c*: Agreeableness positively predicts QOL.

*H1d*: Conscientiousness positively predicts QOL.

*H1e*: Openness positively predicts QOL.

### The mediating role of social support

Social support refers to the individual’s ability to adapt to and face difficulties by interacting with the social environment to obtain different levels of support (Lyrakos, 2012). Social support usually comes from important people, such as family members, friends, and partners (Attar-Schwartz et al., 2019). Important people provide personal substantive or emotional help to enable them to face pressure and negative events and complete tasks. When an individual has a good social support and a close social support network, it means that when he is facing difficulties, he can realize that he can get a lot of resources and assistance to tide over the difficulties smoothly (Guo et al., 2020). When an individual has a healthy physical and mental state and a good sense of well-being, his satisfaction with life is also high. Social support can also help individuals buffer against different pressures in work and life and help them adapt. People with high social support have stable and good interpersonal relations and social interaction (Belayneh et al., 2018). Even if they encounter bottlenecks, they can be sure that they are loved and cared for. When individuals face stressful events in life with such a belief, they can play a buffer role, slow down the impact on individuals, and have the power to face challenges and solve problems.

Ecological systems theory suggests that social factors in the macro-system affect the development of people (Bronfenbrenner, 1979). As one of the most significant macro-system in the outermost system of the social environment, social support can significantly impact the QOL (Wang, 2004). The large body of research on social support and physical and mental health shows that social support is closely related to the QOL (Gao et al., 2022). Good social support is beneficial to health, while malignant social relations damage physical and mental health. Social support has a buffering effect on stress and is important for maintaining a good mood. Some studies show that social support affects the QOL of patients with cognitive disorders (Afunugo and Rafael, 2019), and improved social support may enhance the life quality of lung cancer patients (Hofman et al., 2021). The higher the social support level of the elderly, the better the QOL (Moghadam et al., 2020), patients with breast cancer (Zhang et al., 2017), and hemodialysis patients (Alexopoulou et al., 2016).

The social support theory proposes that the influential factors for social support include personal, improvement, and environmental factors. The personality traits in personal factors influenced the degree of social support (Schoch et al., 2018). Empirical studies have found that personality can affect individuals’ perception of social support to a certain extent (Li et al., 2005). Individuals with different personality traits have different perceived levels of social support (Liu and Huang, 2010). Research results based on diverse objects suggest that higher agreeableness can promote the social support perceived by the individual (Wang and Gen, 2016; Yu et al., 2021). Conscientiousness (Barańczuk, 2019), openness (Bao et al., 2019), and extraversion (Olawa and Idemudia, 2020) have a significant positive correlation with social support. Neuroticism has a negative correlation with social support (Olawa and Idemudia, 2020).

From the above, social support can affect the QOL of PWD, but personality directly affects social support and the QOL of PWD. Therefore, this study hypothesized that personality traits indirectly influence the QOL of PWD through social support. The present study proposed the following hypotheses:

*H2a*: Social support may mediate between neuroticism and QOL.

*H2b*: Social support may mediate between extraversion and QOL.

*H2c*: Social support may mediate between agreeableness and QOL.

*H2d*: Social support may mediate between conscientiousness and QOL.

*H2e*: Social support may mediate between openness and QOL.

## Materials and methods

### Participants and procedures

The sample size was determined, using Raosoft sample size calculator,^1^ based on a margin of error of 5%, confidence level of 90%, population size of 20,000, and a response distribution of 50%. The calculated sample size was 267. The people with disabilities refer to those with visual, hearing, speech, or physical disabilities; mental retardation; mental disorder; multiple disabilities; and/or other disabilities. The questionnaire was completed by the personnel who meet all of the following inclusion criteria:

1. Recruit participants from individuals living with various types of disability.
2. The participants were fully informed of the relevant aspects of the survey, including its aim and methodology of the survey.
3. All subjects participated in this study voluntarily, who had normal language organization and cognitive skills.

This study used the random sampling method to select subjects from 29 provinces and cities of China. In total, 389 questionnaires were distributed, and 358 valid questionnaires were recovered, with an effective rate of 92.03%. Among the 358 respondents, 12 (3.35%) participants were under 19 years old, 218 (60.89%) were between 19 and 59, and 128 (35.76%) were over 59; 165 (46.09%) were male and 193 (53.91%) were female; 51 (14.25%) were single, 218 (60.89%) were married, 43 (12.01%) were divorced, and 46 (12.85%) were widowed; and 61 (17.04%) were visual disabilities, 53 (14.80%) were hearing disabilities, 19 (5.31%) were speech disabilities, 78 (21.79%) were physical disabilities, 13 (3.63%) were mental retardation, 49 (13.69%) were mental disorder, 74 (20.67%) were multiple disabilities, and 11 (3.07%) were other disabilities (Table 1).

**Table 1:** Sample characteristics (*N* = 358).

Characteristics of participants	Variables	Number (%)
Age in years	<19	12 (3.35%)
	19–59	218 (60.89%)
	>59	128 (35.76%)
Gender	Male	165 (46.09%)
	Female	193 (53.91%)
Marital status	Single	51 (14.25%)
	Married	218 (60.89%)
	Divorced	43 (12.01%)
	Widowed	46 (12.85%)
Employment	Students	61 (17.04%)
	Serving personnel	64 (17.88%)
	Retirees	63 (17.60%)
	No fixed occupation	170 (47.49%)
Disability type	Visual disabilities	61 (17.04%)
	Hearing disabilities	53 (14.80%)
	Speech disabilities	19 (5.31%)
	Physical disabilities	78 (21.79%)
	Mental retardation	13 (3.63%)
	Mental disorder	49 (13.69%)
	Multiple disabilities	74 (20.67%)
	Other disabilities	11 (3.07%)
Permanent residence	Urban	164 (45.81%)
	Rural	194 (54.19%)

### Measures

#### Big five personality

The Big Five Inventory-10 (BFI-10; Rammstedt and John, 2007) was used to measure the big five personality traits of disabled people. Yu et al. (2016) revised the scale and formed a simplified version of the Big Five Personality Scale in line with the actual situation in China. This scale contained 10 items, categorized into five dimensions: neuroticism, extraversion, agreeableness, conscientiousness, and openness. Each dimension comprises 2 items, and one of the questions was reverse scored in every dimension. There is a reverse scoring for questions 1, 3, 4, 5, and 7. All items were scored on a five-point Likert scale. The total score of each dimension was calculated separately, and a higher score indicates a more intense personality trait. The internal consistency coefficients of the five dimensions were 0.72, 0.76, 0.71, 0.74, and 0.79. The BFI-10 retained significant levels of validity and reliability (Rammstedt and John, 2007).

#### Quality of life

The QOL of disabled people was evaluated using the European Quality of Life Five Dimension Five Level (EQ-5D-5L; Brooks, 1996) developed by the Euro QOL Group. Luo et al. (2013) translated the questionnaire into Chinese. The EQ-5D-5L was one of the versions of EQ-5D and consisted of a short descriptive system questionnaire and a visual analog scale (EQ VAS). It was categorized into 5 dimensions: Mobility(MO), Self-care(SC), Usual Activities(UA), Pain/Discomfort(PD), and Anxiety/Depression(AD). Each dimension has five response level, with the options ranging from 1 to 5. The digits applied to each dimension are combined in a five-digit number to describe the respondent’s health state. For further statistical analysis, the composite EQ-5D health state scores were converted into utility scores using the EQ-5D-5L Value Set for China. The EQ-5D-5L was proved to have adequate reliability and validity (Herdman et al., 2011; Boczor et al., 2019).

#### Social support

Social support was assessed using the Chinese version of the Perceived Social Support Scale (PSSS) (Jiang, 2001). The PSSS was translated from the Perceived Social Support Scale (Zimet et al., 1988). This scale contained 12 items, divided into two dimensions: support within the family and support outside the family. (e.g., “I can speak with my family about my problems”; “I can speak with my friends about my problems”). All items were rated on a 7-point Likert scale. Higher overall scores indicate greater levels of social support from people perceived. The internal consistency coefficients of the total scale and subscales were 0.94, 0.88, and 0.92. PSSS was proved to show good validity and reliability in previous studies (Jiang, 2001).

### Procedure and statistical analyses

Normality of data was tested using D’Agostino and Pearson’s normality test and Shapiro–Wilk normality test. SPSS 24.0 was used for the descriptive statistical analysis and correlation analysis. Mplus version 7.2 was used to examine the mediating effect of social support on the relationship between neuroticism, extraversion, agreeableness, conscientiousness, openness, and QOL of PWD. Missing data were handled using full-information maximum likelihood estimation, and the significance of paths was verified by bootstrapping analysis with 5,000 replicates.

## Results

### The common method bias examination

Using self-reporting methods to collect data is likely to produce common method bias. According to the recommendations from Zhou Hao and Long Lirong, we took control measures in the test, such as using reverse presentation for some entries. Before analyzing data, the common method bias was evaluated by using the Harman single-factor test. As a result, there were 6 factors with eigenvalues greater than 1, which explained 66.87% of the variance. The first factor accounted for 35.50% of the total variance, which was less than the critical value of 40% (Podsakoff et al., 2003). Thus, there was no serious problem of common method bias in this study.

### Descriptive statistics and correlation analysis

Pearson’s correlation analysis (Table 2) showed that neuroticism was negatively correlated with extraversion (*r* = −0.185*, p* < 0.001), agreeableness (*r* = −0.181*, p* < 0.001), conscientiousness (*r* = −0.175*, p* < 0.001), openness (*r* = −0.114*, p* <0.05), social support (*r* = −0.120*, p* < 0.05), and QOL (*r* = −0.186*, p* < 0.001). QOL was positively correlated with social support (*r* = 0.402*, p* <0.001), extraversion (*r* = 0.324*, p* < 0.001), agreeableness (*r* = 0.474*, p* < 0.001), conscientiousness (*r* = 0.482*, p* < 0.001), and openness (*r* = 0.498*, p* < 0.001). Social support was positively correlated with extraversion (*r* = 0.251*, p* < 0.001), agreeableness (*r* = 0.307*, p* < 0.001), conscientiousness (*r* = 0.370*, p* < 0.001), and openness (*r* = 0.304*, p* < 0.001). Extraversion was positively correlated with agreeableness (*r* = 0.113*, p* < 0.05), conscientiousness (*r* = 0.345*, p* < 0.001), and openness (*r* = 0.238*, p* < 0.001). Agreeableness was positively correlated with conscientiousness (*r* = 0.508*, p* < 0.001) and openness (*r* = 0.361*, p* < 0.001). And a significant positive relationship was observed between conscientiousness and openness (*r* = 0.379*, p* < 0.001).

**Table 2:** Correlations, means, and standard deviations of all study variables.

Variable	*M*	*SD*	1	2	3	4	5	6	7	8	9
1. Neuroticism	2.862	0.790	1								
2. Extraversion	3.064	0.815	−0.185^***^	1							
3. Agreeableness	3.302	0.889	−0.181^***^	0.113^*^	1						
4. Conscientiousness	3.358	0.954	−0.175^***^	0.345^***^	0.508^***^	1					
5. Openness	3.068	0.934	−0.114^*^	0.238^***^	0.361^***^	0.379^***^	1				
6. Support within the family	4.835	1.184	−0.134^*^	0.239^***^	0.282^***^	0.369^***^	0.243^***^	1			
7. Support outside the family	4.728	1.060	−0.109^*^	0.235^***^	0.294^***^	0.338^***^	0.310^***^	0.758^***^	1		
8. Social support	4.764	1.038	−0.120^*^	0.251^***^	0.307^***^	0.370^***^	0.304^***^	0.896^***^	0.969^***^	1	
9. Quality of life	0.748	0.191	−0.186^***^	0.324^***^	0.474^***^	0.482^***^	0.498^***^	0.348^***^	0.397^***^	0.402^***^	1

* *p* < 0.05.

** *p* < 0.01.

*** *p* < 0.001.

### The mediation role of social support between big five personality and quality of life

Structural equation modeling was used to test the study hypotheses. The model estimation method was the maximum likelihood estimation method. The structural equation analysis was performed with neuroticism, extraversion, agreeableness, conscientiousness, and openness as the predictors, employment and permanent residence as control variables, social support as the mediator, and QOL as the outcome variable. The significance test was estimated using the bias-corrected bootstrap method and repeated 5,000 times after putting it back.

**Figure 1:**
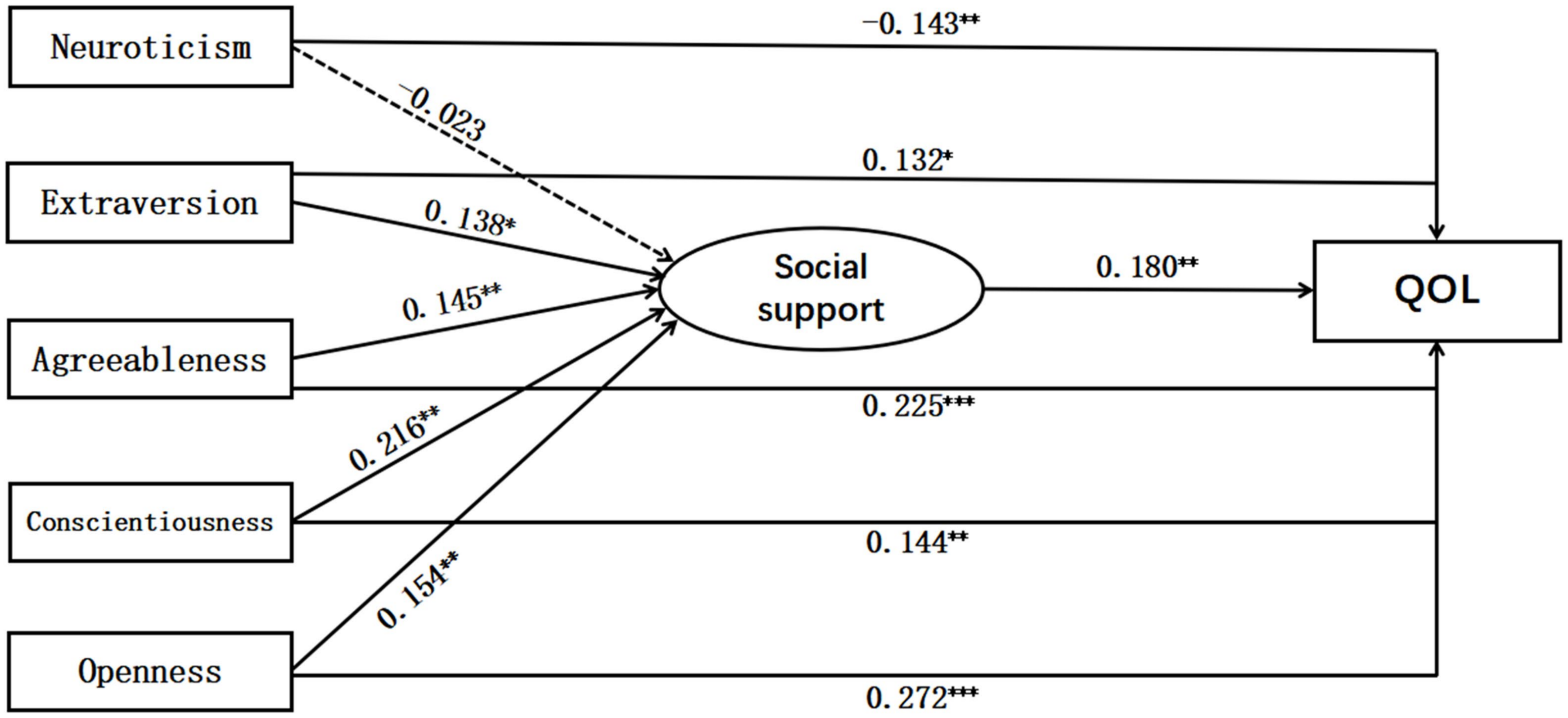
The path coefficients of the model.

The results of the hypothesized model showed good overall model fit indices: *χ*2 = 6.299, *df* = 5, RMSEA = 0.047 [90% CI: 0.001, 0.093], CFI = 0.995, TLI = 0.980, SRMR = 0.013 (Figure 1).

Further examination of the model used a bias-corrected bootstrap method. Results (see Table 3) showed that the 95% confidence interval of other mediation effects did not contain 0, except for the mediating effect of neuroticism → social support → quality of life. It means other mediation effects were significant, except for the mediating effect of neuroticism → social support → quality of life. Therefore, social support mediated the relationship between extraversion, agreeableness, conscientiousness, openness, and quality of life. And the mediation effects of social support were 0.025, 0.026, 0.039, and 0.028. H2b, H2c, H2d, and H2e are all supported.

**Table 3:** Bootstrap results for each path coefficient of the hypothetical model.

Effects	Model pathway	95% CI	Effect
Direct effect	Neuroticism → quality of life	[0.056, 0.230]	0.143
	Extraversion → quality of life	[0.054, 0.210]	0.132
	Agreeableness → quality of life	[0.136, 0.314]	0.225
	Conscientiousness → quality of life	[0.057, 0.231]	0.144
	Openness → quality of life	[0.193, 0.350]	0.272
Mediating effect	Extraversion → social support → quality of life	[0.000, 0.050]	0.025
	Agreeableness → social support → quality of life	[0.001, 0.051]	0.026
	Conscientiousness → social support → quality of life	[0.007, 0.071]	0.039
	Openness → social support → quality of life	[0.002, 0.054]	0.028

## Discussion

This study investigated the relationship between big five personality and the QOL of PWD and whether this relationship varies with the social support they perceived. The results suggest that social support had a non-significant mediating effect between neuroticism and the QOL of PWD was not significant, which disproved hypothesis H1a. Previous studies have confirmed that neuroticism affects social support (Olawa and Idemudia, 2020; Yu and Hu, 2022) and QOL(Harandi et al., 2020; Dedova et al., 2022), but many of these studies did not choose PWD as research subjects (Masthoff et al., 2007; Poppe et al., 2013). Also, previous studies merely confirmed that neuroticism could directly affect social support or QOL, which does not mean that neuroticism can indirectly influence the QOL of PWD through social support. Neuroticism is a negative personality trait (Judge et al., 1999; Yang et al., 2021) which easily experiences negative emotions such as fear and anxiety (Okbay et al., 2016). The findings indicate that PWD have worse health status than the able-bodied population (Huang et al., 2019). Accordingly, the living conditions of PWD with neuroticism are more complex and harsh. Although social support can improve living conditions outside, neuroticism PWD may have lower hopes for life, so outside social support might not be effective in improving the QOL of neuroticism PWD. From this, to improve the QOL of neurotic PWD, we cannot rely on single social support but also need to take other necessary measures. Such as regular exercise and good living habits are irreplaceable by any medications and medical treatment (Xu and Fei, 2019).

The present results show that social support has significant mediating effects between extraversion, agreeableness, conscientiousness, openness, and QOL, which verified hypotheses H1b, H1c, H1d, and H1e. According to the social support theory, personality traits have a crucial impact on social support (Mai et al., 2021), and sufficient social support is beneficial to motivate them to face life more positively (Lee et al., 2016). A positive attitude means they are hopeful for their future, and improving self-thought can improve the QOL of people (Takeda et al., 2019). Moreover, PWD cannot accomplish many things as efficiently as the able-bodied population, so social support is more significant for PWD. Upon inquiry, more than 70% of the economic income of PWD in China comes from the state and collectives, about 20% comes from charities, and not exceed 10% comes from their families and relatives. Better social support can improve the standard of living of PWD and provide them with better medical security to increase their QOL.

### Advantages and limitations

The advantages of the study are as follows. Firstly, this is a China-wide cross-sectional investigation, and the selected sample is representative to a certain extent, which makes the research results more credible. Secondly, this study verified the relationship between big five personality and the QOL of PWD. Neuroticism negatively influenced the QOL, while the other four personalities could positively affect the QOL. Thirdly, positive personality traits may help PWD perceive social support. They received help that they perceived, thereby improving their QOL. Therefore, we will take measures to increase social support in the future to increase the QOL of PWD. Fourth, the study found that the mediating influence of social support on neuroticism and the QOL was not significant. It shows that social support alone cannot improve the QOL of disabled people with neuroticism. Thus, we should pay more attention to such populations with disabilities, and other approaches are required to improve their QOL. Finally, the mediation model indicated that considering not only individual but also environmental factors is required to propose systematic and comprehensive programs which can improve the QOL of PWD.

The present study had some limitations, and future studies should address them. First, this study was a cross-sectional study. Although it is advantageous for analyzing the specific relationship between different variables, the lack of longitudinal data is not conducive to inspecting variables in this study, especially testing the direction between them. In subsequent studies, we can investigate whether the relationship between the big five personality, QOL of PWD, and social support will change over time. Second, the data in the present study which collected from self-reports of PWD. The self-report can reflect individuals’ physical and mental conditions, but the self-report is prone to measurement error. In the follow-up study, we can consider obtaining information from all sides to reduce measurement error. Third, openness is a western abstract concept and may be hard to be realized for Chinese, especially elderly people with low income and low academic status. The participants may not express their actual openness status correctly.

## Conclusion

The mediating effect of social support on the relationship between neuroticism and the QOL of PWD was not significant. Social support significantly mediated the relationship between extraversion, agreeableness, conscientiousness, openness, and QOL. Overall, positive personality traits (extraversion, agreeableness, conscientiousness, openness) in the big five personality of PWD could increase their QOL by Perceiving social support. But social support could not significantly mediate the relationship between neuroticism and the QOL of PWD. These new findings suggest that combining individual factors (personality) and environmental factors (social support) can improve the QOL of PWD.

## Data availability statement

The original contributions presented in the study are included in the article/supplementary material, further inquiries can be directed to Lin Cai, 292504017@qq.com.

## Ethics statement

The study was approved by the Ethics Committee of Jinan University, China (statement JNUKY-2021-018). The patients/participants provided their written informed consent to participate in this study.

## Author contributions

LC and JH designed the research and wrote the manuscript. YW planned and conducted the data collection. LC analyzed the data and revised the manuscript. All authors contributed to the article and approved the submitted version.

## Funding

This study was supported by Industry and Education Cooperation Program of the Ministry of Education (no. 220503924164635) and the project of teaching reform in Higher Education of Sichuan Province of China (no. JG2021-1492).

## Conflict of interest

The authors declare that the research was conducted in the absence of any commercial or financial relationships that could be construed as a potential conflict of interest.

## Publisher’s note

All claims expressed in this article are solely those of the authors and do not necessarily represent those of their affiliated organizations, or those of the publisher, the editors and the reviewers. Any product that may be evaluated in this article, or claim that may be made by its manufacturer, is not guaranteed or endorsed by the publisher.

## Footnotes

^1^ http://www.raosoft.com/samplesize.html
